# Prevalence of Syndecan-1 (CD138) Expression in Different Kinds of Human Tumors and Normal Tissues

**DOI:** 10.1155/2019/4928315

**Published:** 2019-12-23

**Authors:** Simon Kind, Christina Merenkow, Franziska Büscheck, Katharina Möller, David Dum, Viktoria Chirico, Andreas M. Luebke, Doris Höflmayer, Andrea Hinsch, Frank Jacobsen, Cosima Göbel, Sören Weidemann, Christoph Fraune, Christina Möller-Koop, Claudia Hube-Magg, Till S. Clauditz, Ronald Simon, Guido Sauter, Waldemar Wilczak, Ahmed Abdulwahab Bawahab, Jakob R. Izbicki, Daniel Perez, Andreas Marx

**Affiliations:** ^1^Institute of Pathology, University Medical Center Hamburg-Eppendorf, 20246 Hamburg, Germany; ^2^Institute of Pathology, University of Jeddah, 21589 Jeddah, Saudi Arabia; ^3^General, Visceral and Thoracic Surgery Department and Clinic, University Medical Center Hamburg-Eppendorf, 20246 Hamburg, Germany; ^4^Institute of Pathology, Jakob-Henle-Straße 1 90766 Fürth, Germany

## Abstract

Syndecan-1 (CD138) is a transmembrane proteoglycan known to be expressed in various normal and malignant tissues. It is of interest because of a possible prognostic role of differential expression in tumors and its role as a target for indatuximab, a monoclonal antibody coupled with a cytotoxic agent. To comprehensively analyze CD138 in normal and neoplastic tissues, we used tissue microarrays (TMAs) for analyzing immunohistochemically detectable CD138 expression in 2,518 tissue samples from 85 different tumor entities and 76 different normal tissue types. The data showed that CD138 expression is abundant in tumors. At least an occasional weak CD138 immunostaining could be detected in 71 of 82 (87%) different tumor types, and 58 entities (71%) had at least one tumor with a strong positivity. In normal tissues, a particularly strong expression was found in normal squamous epithelium of various organs, goblet and columnar cells of the gastrointestinal tract, and in hepatocytes. The highly standardized analysis of most human cancer types resulted in a ranking order of tumors according to the frequency and levels of CD138 expression. CD138 immunostaining was highest in squamous cell carcinomas such as from the esophagus (100%), cervix uteri (79.5%), lung (85.7%), vagina (89.7%) or vulva (73.3%), and in invasive urothelial cancer (76.2%). In adenocarcinomas, CD138 was also high in lung (82.9%) and colorectal cancer (85.3%) but often lower in pancreas (73.3%), stomach (54.2% in intestinal type), or prostate carcinomas (16.3%). CD138 expression was usually low or absent in germ cell tumors, sarcomas, endocrine tumors including thyroid cancer, and neuroendocrine tumors. In summary, the preferential expression in squamous cell carcinomas of various sites makes these cancers prime targets for anti-CD138 treatments once these might become available. Abundant expression in many different normal tissues might pose obstacles to exploiting CD138 as a therapeutic target, however.

## 1. Introduction

Syndecan-1 (CD138) is one of four members of the syndecan family. It is a cell surface protein consisting of three structural domains, one of which is extracellular and binds heparin sulfates and chondroitin sulfates [1]. Syndecan-1 has relevance for cell-cell and cell-matrix interactions [1]. It is involved in the regulation of cell proliferation, migration, and the organization of the cytoskeleton [1]. In normal tissues, CD138 is known to be expressed on plasma cells and various epithelial cell types.

CD138 expression in cancer is of potential clinical interest as specific drugs targeting CD138 are currently being evaluated in clinical trials. In a phase II trial on plasmocytoma, clinical efficacy and low side effects have been reported [2, 3]. In preclinical studies, these antibodies also showed efficacy against triple negative breast cancer and melanoma [4, 5]. If anti-CD138 therapies should prove successful, other CD138-positive cancer types might as well benefit from such treatments.

Altered CD138 expression has been described in various malignant tumors. For example, overexpression of CD138 has been reported in breast, urinary bladder, gallbladder, pancreatic, ovarian, endometrial, and prostate cancer [1]. In other cancer types, such as lung, head/neck, gastric, renal, and colorectal cancer, CD138 expression was found to be reduced as compared to adjacent normal epithelium [1]. In several of these tumor types, either reduced or increased CD138 expression was linked to unfavorable tumor phenotype and poor patient prognosis [6–9]. Previous studies on CD138 in cancer have applied various different reagents and protocols for their immunohistochemical staining. It is probably because of this that the existing literature is highly discrepant with respect to the prevalence of CD138 expression in different tumor types. For example, the range of the reported CD138 positivity ranges from 26% [10] to 100% [11] in urinary bladder cancer, from 23% [10] to 89% [12] in squamous lung cancer, from 33% [13] to 100% [14] in breast cancer, from 50.5% [15] to 87% [10] in squamous cell carcinoma of the esophagus, and from 24.7% [16] to 89.7% [17] in squamous cell carcinoma of the cervix.

Given these heterogeneous data, the existing literature does not easily allow to determine these cancer types, where CD138 plays a particularly important role. To compare the prevalence and intensity of CD138 expression between tumor entities and to identify these cancer types that might be optimal candidates for anti-CD138 drugs, we thus analyzed more than 2500 cancers and 76 normal tissues using one standard protocol. For this purpose, a multitumor tissue microarray (TMA) was used containing up to 50 different tumors from 85 different tumor types and subtypes. The results of our study identify a broad range of highly CD138-expressing tumor entities.

## 2. Materials and Methods

### 2.1. Tissue Microarrays (TMAs)

We used two different sets of preexisting TMAs to study CD138 expression in normal human and cancerous human tissues. The first TMA was composed of one sample of 76 different normal tissue types (608 samples on one slide). The second TMA contained a total of 3,642 primary tumors from 85 tumor types and subtypes. The samples were distributed among 7 different TMA blocks (containing between 414 and 522 samples). The composition of the TMA is described in Table 1 in Results. All samples were derived from the archives of the Institute of Pathology, University Hospital of Hamburg (Hamburg, Germany). Each TMA block contains an identical standard control section with 40 normal and tumor tissue spots in order to control for possible slide-to-slide variability of the immunostaining. Tissues were fixed in 4% buffered formalin and then embedded in paraffin. TMA tissue spot diameter was 0.6 mm. All works were compliant with the Helsinki Declaration. Informed consent was not necessary.

### 2.2. Immunohistochemistry

Freshly cut TMA sections were immunostained on one day and in one experiment. Slides were deparaffinized and exposed to heat-induced antigen retrieval for 5 minutes in an autoclave at 121°C in pH 9 Dako Target Retrieval Solution buffer. Primary antibody specific for total Syndecan-1 (mouse monoclonal antibody, clone JASY1, Dianova, Hamburg, Germany, dilution 1 : 200) was applied at 37°C for 60 minutes. Bound antibody was then visualized using the EnVision Kit (Dako, Glostrup, Denmark) according to the manufacturer's directions. For tumor tissues, the percentage of positive epithelial cells was estimated and the staining intensity was semiquantitatively recorded (0, 1+, 2+, and 3+). For statistical analyses, the staining results were categorized into four groups. Tumors without any staining were considered as negative. Tumors with 1+ staining intensity in ≤70% of cells and 2+ intensity in ≤30% of cells were considered weakly positive. Tumors with 1+ staining intensity in >70% of cells, 2+ intensity in 30% to 70%, or 3+ intensity in ≤30% were considered moderately positive. Tumors with 2+ intensity in >70% or 3+ intensity in >30% of cells were considered strongly positive. These categories represent standard cutoffs that others and us have used in numerous IHC studies [18].

## 3. Results

### 3.1. Technical Issues

A total of 2,518 (69%) of the 3,642 tumor tissue samples were interpretable in our TMA analysis. Reasons for analysis failure included a fraction of missing samples or samples lacking unequivocal tumor cells. A sufficient number of samples were analyzable for all 76 normal tissue types enabling a complete normal tissue evaluation.

### 3.2. Syndecan-1 in Normal Tissues

**Figure 1:**
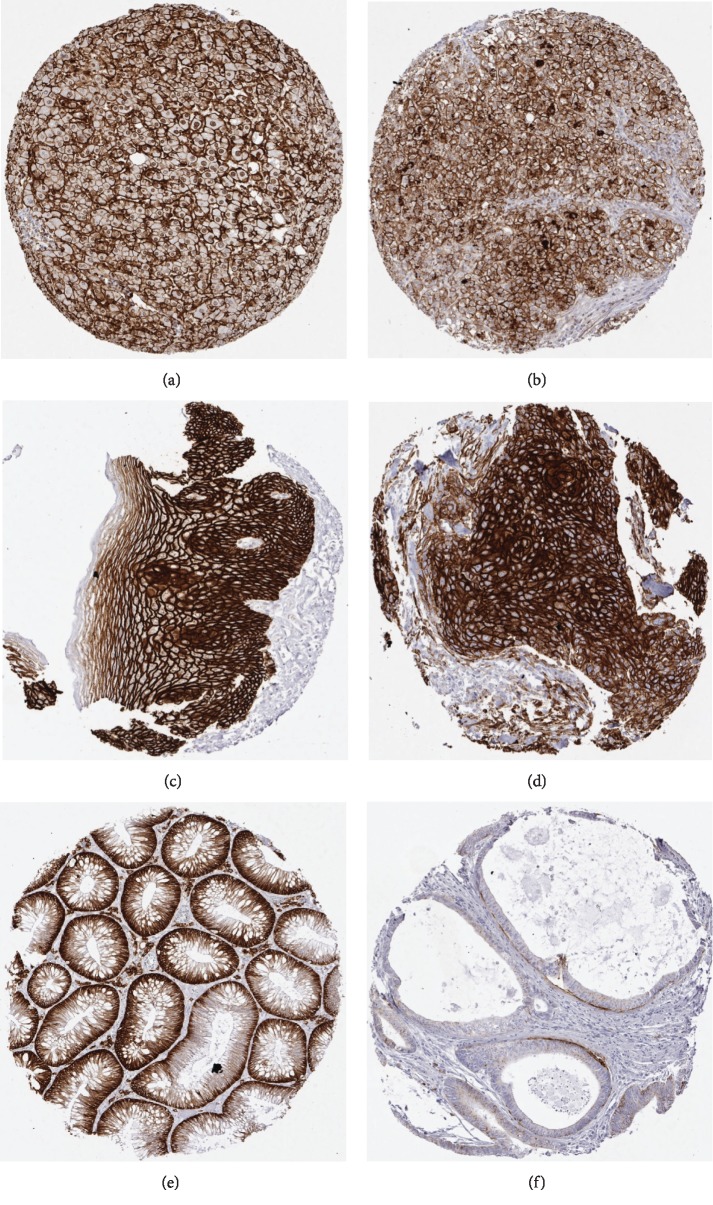
Representative images of CD138 immunostaining in normal and tumorous tissues: (a) normal liver, (b) hepatocellular carcinoma, (c) normal esophagus, (d) squamous cell carcinoma of the esophagus, (e) colon adenoma, and (f) colon carcinoma.

All positive CD138 immunostainings in normal tissues are summarized in Table 2. CD138 was abundantly expressed, mostly in various epithelial cell types. A particularly strong expression of CD138 was observed in squamous epithelial cells of various organs (Figure 1(a)), goblet cells of the gastrointestinal tract (Figure 1(b)), columnar cells in the gall bladder (Figure 1(c)), and hepatocytes (Figure 1(d)). No CD138 staining was detected in the following tissues: aorta/intima, aorta/media, heart (left ventricle), skeletal muscle, skeletal muscle/tongue, myometrium, muscular wall appendix, esophagus, stomach, ileum, colon descendens, kidney pelvis and urinary bladder, penis (glans/corpus spongiosum), ovary (stroma), fat tissue (white), spleen, thymus, ovary (corpus luteum), ovary (follicular cyst), thyroid, cerebellum, cerebrum, pituitary gland (posterior lobe), pituitary gland (anterior lobe), and bone marrow.

### 3.3. CD138 in Tumorous Tissues

Immunostaining was predominantly membranous but sometimes also in the cytoplasm. Occasional stroma staining also occurred but was disregarded for this analysis. CD138 positivity was seen in 1,118 of 2,518 analyzable tumors (Table 1). CD138 immunostaining was considered weak in 330 (13%), moderate in 226 (9%), and strong in 562 tumors (22%). Representative tumor tissue spots with CD138 expression are shown in Figures 1(e) and 1(h) . At least some CD138 expression could be detected in 75 of 85 (88%) of our tumor categories, including 60 (71%) categories where at least one tumor showed a strong positivity (Table 1). The tumor types where some CD138 was seen in all analyzed cases included basal cell adenoma, colon adenoma, squamous carcinoma of the esophagus, granular cell tumor, and ovarian Brenner tumor. A particularly significant CD138 expression was also detected in anal carcinoma (90.9%), squamous carcinoma of the skin (92.9%), hepatocellular carcinoma (97.7%), phyllodes carcinoma of the breast (94.4%), and in Warthin tumor of the parotis (92.7%). Tumor types with a particularly low or absent CD138 immunostaining included testicular germ cell tumors, several sarcomas, melanoma, malignant mesothelioma, and small cell urinary bladder carcinoma.

## 4. Discussion

The results of this study provide a comprehensive overview on Syndecan-1 expression in human tumors. The data show that—across all organs of origin—squamous cell (and urothelial) carcinomas are particularly prone to express Syndecan-1, often at high levels. Even though adenocarcinomas derived from the colon and the lung are also high expressers, CD138 immunostaining appears to be generally less intense and less frequent in adenocarcinomas. This is best visible in organs where both adenocarcinomas and squamous cell carcinomas occur as in the uterine cervix and in the esophagus. The squamous cell predominance of CD138 expression even becomes apparent in cancers with identical pathogenesis such as cervical cancer, oral cancer, or squamous cell carcinoma of the anus, which are often human papilloma virus associated. Clinically important cancer types with low to intermediate frequencies and levels of CD138 expression include cancers of the kidney and of the endometrium while low frequencies of positivity were found in prostate cancer, endocrine tumors including thyroid cancer, and neuroendocrine tumors as well as germ cell cancers. Despite some outliers, our data are largely consistent with the literature. Several other investigators had earlier described particularly high levels of CD138 expression in squamous cell cancer [17, 19, 20].

**Figure 2:**
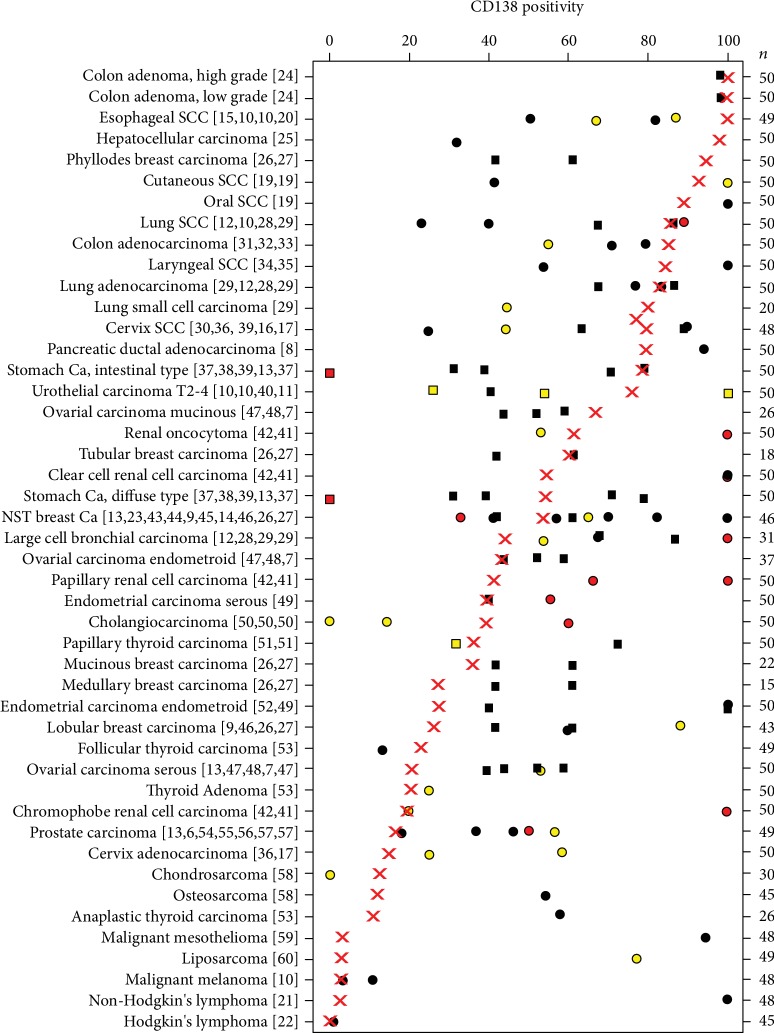
Overview of CD138 expression in tumorous tissue from various studies ranked according to the present study (red cross), dots for studies with subtype specific data, squares for studies with no subtype specific data, and color code indicates the number of tumors included in earlier studies: red: <10, yellow: 10–25, and black: >25.

The standardized assessment of 85 different tumor types and subtypes enabled us to define a ranking order with respect to the level of CD138 expression in cancer. We believe these data are particularly helpful for these tumor types for which previous data had been partially discrepant. The study also provided information on a number of relevant tumor types for which CD138 data were lacking so far. These for example include squamous cell carcinoma of the vulva and the anal canal, adenocarcinoma of the esophagus, seminoma, embryonal carcinoma and yolk sac tumor of the testis, small cell bladder cancer, neuroendocrine tumors of the pancreas, pheochromocytoma, thymoma, gastrointestinal stroma tumor (GIST), angiosarcoma, and leiomyosarcoma. Moreover, subtype-specific data were obtained for several tumor types, for which previous analyses were performed on tumor cohorts with less detailed information on tumor morphology such as urothelial carcinoma, breast, endometrium, and ovarian cancers. These data thus represent another example of the suitability of TMAs composed of samples of many different tumor types and normal tissues for comprehensively characterizing a biomarker or antibody (demonstration of our data compared to previous studies is shown in Figure 2) [6–13, 15–17, 19–60].

Various earlier studies have suggested a link to poor patient outcome for either increased or decreased Syndecan-1 levels [1]. CD138 protein can have tumor suppressor and tumor-promoting functions which depend on the tumor [61]. Our tumor cohort was devoid of any tumor stage or clinical outcome information. Indirect evidence for a variable role of differential CD138 expression for tumor progression comes from the comparison of related tumor subtypes, however. For example, the lower expression levels of CD138 in colorectal adenocarcinoma and in invasive urothelial carcinoma (pT2-4) as compared to colon adenomas and noninvasive bladder tumors (pTa) argues for a loss of CD138 paralleling tumor progression in these tumors. The lower expression of CD138 in chromophobe renal carcinoma as compared to its benign counterpart oncocytoma may also be viewed as an argument for CD138 downregulation being linked to tumor aggressiveness in these kidney cancers derived from the distal nephron tubulus. However, higher levels of CD138 expression in the cortical adrenal carcinoma than in the adrenal adenoma suggest that increased CD138 levels may accompany progression in these tumors.

CD138 is a membrane protein and as such a potential target for antibody therapeutics. There are efforts to develop a suitable therapy for CD138-positive cancers. CD138 was shown to be overexpressed on the surface of multiple myeloma cells which is used in a preclinical study for an antitumoral therapy with indatuximab, a monoclonal antibody coupled with a cytotoxic agent, which is currently evaluated in preclinical studies on plasmacytoma [3] and triple-negative breast cancers in combination with other drugs [4]. Based on our data, squamous cell carcinomas, irrespective of their site of origin, emerge as further possible candidates for anti-CD138 therapy once such a treatment should prove to be efficient and become available. The abundant expression of CD138 in various normal tissues including squamous epithelium from various different organs identifies various sites where potential side effects of these therapies might emerge.

CD138 expression analysis is currently used in routine diagnostic pathology to distinguish and quantitate plasma cells, for example, in the bone marrow and in endometrial biopsies where the presence of plasma cells indicates chronic endometritis. Apart from two possible exceptions, our data provide little evidence for Syndecan-1 expression analysis providing diagnostic clues in difficult diagnostic situations. The low expression in mesothelioma as compared to the high prevalence of strong expression in pulmonary adenocarcinoma suggests that Syndecan-1 could be potentially added to the long list of antibodies that help to distinguish these tumor entities. A low frequency of Syndecan-1 expression (10%) has recently also been described for peritoneal mesothelioma [62]. Moreover, CD138 was markedly higher in hepatocellular carcinoma as compared to cholangiocellular carcinoma of the liver. However, other antibodies as, for example, arginase or BSEP are better separators of these tumor entities [63–65].

It is a limitation of this study that immunohistochemistry approaches, especially when using bright-field visualization, are not optimal for protein quantification. Importantly, the lack of immunostaining does not exclude a biologically relevant CD138 expression in “negative” normal or neoplastic cells. Every protocol defines a detection threshold below of which tissues are considered negative. Above this detection limit, the staining intensity enables a certain quantification of proteins but this is limited by a maximum intensity staining which cannot become discernably stronger in case of even higher protein expression levels. Moreover, occasional stromal staining had been disregarded in our study, although others and us have shown that there is evidence for a clinically relevant role of CD138 expression in the tumor-associated stroma [23, 57, 66]. However, stroma staining is infrequent and would require larger sample numbers per cancer type for a meaningful analysis.

In summary, this study provides a comprehensive overview on CD138 expression in human tumors. The preferential expression in squamous cell carcinomas of various sites makes these cancers prime targets for anti-CD138 treatments once these might become available. Abundant expression in many different normal tissues might pose obstacles to exploiting CD138 as a therapeutic target, however.

## Figures and Tables

**Table 1:** CD138 expression in different tumorous tissues.

			CD138 immunhistochemistry results (%)					
	Entity	*n* on TMA	*n* analyzable	Negative %	Weak %	Moderate %	Strong %	Positive %
Tumors of the skin	Pilomatrixoma	35	30	63.3	26.7	10.0	0.0	36.7
	Basalioma	48	41	2.4	7.3	7.3	82.9	97.6
	Epidermal nevus	29	19	100.0	0.0	0.0	0.0	0.0
	Cutaneous squamous cell carcinoma	50	42	7.1	21.4	9.5	61.9	92.9
	Malignant melanoma	48	39	97.4	2.6	0.0	0.0	2.6
	Merkel cell carcinoma	46	42	69.0	21.4	2.4	7.1	31.0

Tumors of the airway	Laryngeal squamous cell carcinoma	50	44	15.9	31.8	11.4	40.9	84.1
	Oral squamous cell carcinoma	50	45	11.1	22.2	20.0	46.7	88.9
	Lung squamous cell carcinoma	50	21	14.3	14.3	9.5	61.9	85.7
	Large cell bronchial carcinoma	31	25	56.0	32.0	4.0	8.0	44.0
	Lung adenocarcinoma	50	35	17.1	34.3	20.0	28.6	82.9
	Lung in situ pulmonary adenocarcinoma	6	15	80.0	20.0	0.0	0.0	20.0
	Lung small cell carcinoma	20	5	20.0	20.0	40.0	20.0	80.0
	Malignant mesothelioma	48	35	97.1	2.9	0.0	0.0	2.9
	Parotid gland pleomorphic adenoma	50	37	21.6	10.8	18.9	48.6	78.4
	Parotid gland Warthin tumor	49	41	7.3	29.3	22.0	41.5	92.7
	Salivary gland Basal cell adenoma	15	15	0.0	33.3	33.3	33.3	100.0

Gynecological tumors	Vagina squamous cell carcinoma	48	29	10.3	27.6	24.1	37.9	89.7
	Vulva squamous cell carcinoma	50	30	26.7	13.3	33.3	26.7	73.3
	Cervix squamous cell carcinoma	50	39	20.5	23.1	25.6	30.8	79.5
	Cervix adenocarcinoma	50	41	85.4	14.6	0.0	0.0	14.6
	Endometrial carcinoma endometrioid	50	44	72.7	13.6	11.4	2.3	27.3
	Endometrial carcinoma serous	50	33	60.6	9.1	18.2	12.1	39.4
	Uterus stromal sarcoma	12	10	90.0	0.0	10.0	0.0	10.0
	Carcinosarcoma	48	47	74.5	12.8	8.5	4.3	25.5
	Ovarian carcinoma endometrioid	37	30	56.7	16.7	6.7	20.0	43.3
	Ovarian carcinoma serous	50	39	79.5	10.3	7.7	2.6	20.5
	Ovarian carcinoma mucinous	26	21	33.3	19.0	0.0	47.6	66.7
	Brenner tumor	9	6	0.0	0.0	0.0	100.0	100.0
	NST breast carcinoma	46	28	46.4	17.9	7.1	28.6	53.6
	Lobular breast carcinoma	43	27	74.1	11.1	7.4	7.4	25.9
	Medullary breast carcinoma	15	11	72.7	9.1	9.1	9.1	27.3
	Tubular breast carcinoma	18	10	40.0	10.0	10.0	40.0	60.0
	Mucinous breast carcinoma	22	14	64.3	28.6	0.0	7.1	35.7
	Phyllodes breast tumor	50	18	5.6	22.2	38.9	33.3	94.4

Gastrointestinal tumors	Colon adenoma, low grade	50	27	0.0	3.7	3.7	92.6	100.0
	Colon adenoma, high grade	50	25	0.0	4.0	4.0	92.0	100.0
	Colon adenocarcinoma	50	34	14.7	35.3	20.6	29.4	85.3
	Small intestine adenocarcinoma	10	4	75.0	25.0	0.0	0.0	25.0
	Stomach carcinoma diffuse type	50	24	45.8	16.7	8.3	29.2	54.2
	Stomach carcinoma intestinal type	50	28	21.4	42.9	14.3	21.4	78.6
	Esophageal adenocarcinoma	50	33	30.3	27.3	12.1	30.3	69.7
	Esophageal squamous cell carcinoma	49	33	0.0	12.1	9.1	78.8	100.0
	Anal squamous cell carcinoma	50	22	9.1	18.2	9.1	63.6	90.9
	Cholangiocarcinoma	50	23	60.9	17.4	8.7	13.0	39.1
	Hepatocellular carcinoma	50	44	2.3	15.9	9.1	72.7	97.7
	Pancreatic ductal adenocarcinoma	50	30	26.7	36.7	16.7	20.0	73.3
	Pancreas/papilla adenocarcinoma	30	17	29.4	17.6	11.8	41.2	70.6
	Pancreatic neuroendocrine tumor	49	28	82.1	3.6	3.6	10.7	17.9
	Gastrointestinal stroma tumor (GIST)	50	37	100.0	0.0	0.0	0.0	0.0

Urogenital tumors	Urothelial carcinoma pTa	50	41	12.2	2.4	4.9	80.5	87.8
	Urothelial carcinoma T2-4	50	42	23.8	4.8	16.7	54.8	76.2
	Small cell urothelial carcinoma	18	18	100.0	0.0	0.0	0.0	0.0
	Clear cell renal cell carcinoma	50	46	45.7	13.0	17.4	23.9	54.3
	Papillary renal cell carcinoma	50	44	59.1	22.7	6.8	11.4	40.9
	Chromophobe renal cell carcinoma	50	42	81.0	9.5	9.5	0.0	19.0
	Renal oncocytoma	50	44	38.6	27.3	20.5	13.6	61.4
	Prostate carcinoma	49	43	83.7	4.7	4.7	7.0	16.3
	Small cell prostate carcinoma	17	11	54.5	27.3	9.1	9.1	45.5
	Seminoma	50	47	100.0	0.0	0.0	0.0	0.0
	Embryonic carcinoma (testis)	50	39	97.4	2.6	0.0	0.0	2.6
	Yolk sac tumor	50	25	92.0	4.0	4.0	0.0	8.0
	Teratoma	50	23	17.4	4.3	8.7	69.6	82.6

Endocrine tumors	Thyroid adenoma	50	45	80.0	13.3	2.2	4.4	20.0
	Papillary thyroid carcinoma	50	36	63.9	8.3	13.9	13.9	36.1
	Follicular thyroid carcinoma	49	44	77.3	9.1	6.8	6.8	22.7
	Medullary thyroid carcinoma	50	29	82.8	3.4	0.0	13.8	17.2
	Anaplastic thyroid carcinoma	26	19	89.5	5.3	0.0	5.3	10.5
	Adrenal adenoma	50	48	72.9	10.4	6.3	10.4	27.1
	Adrenal carcinoma	26	14	35.7	21.4	14.3	28.6	64.3
	Pheochromocytoma	50	32	100.0	0.0	0.0	0.0	0.0
	Neuroendocrine tumor (NET)	50	27	81.5	7.4	7.4	3.7	18.5

Hemic neoplasia	Hodgkin lymphoma	45	43	100.0	0.0	0.0	0.0	0.0
	Non-Hodgkin lymphoma	48	42	97.6	2.4	0.0	0.0	2.4
	Thymoma	29	24	70.8	16.7	8.3	4.2	29.2

Soft tissue tumors	Giant cell-long sheath tumor	45	41	97.6	2.4	0.0	0.0	2.4
	Granular cell tumor	30	24	0.0	12.5	25.0	62.5	100.0
	Leiomyoma	50	41	100.0	0.0	0.0	0.0	0.0
	Leiomyosarcoma	49	46	93.5	0.0	6.5	0.0	6.5
	Liposarcoma	49	34	97.1	2.9	0.0	0.0	2.9
	Angiosarcoma	32	22	90.9	4.5	4.5	0.0	9.1

Bone neoplasm	Osteosarcoma	25	17	88.2	5.9	5.9	0.0	11.8
	Chondrosarcoma	25	8	87.5	0.0	12.5	0.0	12.5

**Table 2:** CD138 expression in normal human tissues.

Organ systems	Cell type (strong staining +++)	Cell type (moderate staining ++)	Cell type (weak staining +)
Urogenital kidney, urinary bladder, prostate, seminal vesicle, epididymis		Tubular cells, collecting duct cells	Urothelial basal/intermediate/umbrella cells, basal cells of the prostate and seminal vesicle, some columnar cells of the epididymis

Gynecology breast, cervix uteri, uterus corpus, placenta (early and mature)	Ectocervical: basal cells	Ectocervical: intermediate cells, superficial endometrial cells, cytotrophoblasts, syncytiotrophoblasts	Breast: excretory duct cells and myoepithelial cells, endocervical: some mucous cells; endometrium: some basalis type cells and some secretory cells, decidual cells

Skin, sebaceous gland		Basal cells, keratocytes, peripheral germinative cells of the sebaceous gland	Sebaceous gland cells

Lip, oral cavity, tonsil	Keratocytes, squamous epithelial cells		

Salivary glands parotid gland, submandibular gland, sublingual gland		Serous cells	Some serous cells, some columnar ductal cells, myoepithelial cells, intralobular duct cells of the submandibular gland, columnar ductal cells of the sublingual gland

Gastrointestinal tract esophagus, stomach, duodenum, ileum, colon descendens, rectum, anal canal and transitional mucosa, appendix vermiformis	Squamous basal cells of the esophagus, keratinocytes, parietal cells and chief cells of the stomach, crypt cells, goblet cells, absorptive cells, basal cells and keratinocytes of the anal skin	Mucous secreting cells and columnar cells of the stomach, Brunner glands	

Gallbladder	Columnar cells, mucous gland cells		

Liver	Hepatocytes		Interlobular bile duct cells

Pancreas			Excretory duct cells

Airway lung, bronchus, sinus paranasal		Basal cells, ciliated cells, goblet cells	Pneumocytes, bronchus glands: basal cells and serous cells, goblet cells of the sinus paranasales

Endocrine adrenal gland, parathyroid		Cortical cells	Oxyphil cells and chief cells of the parathyroid

## Data Availability

The immunohistochemistry data used to support the findings of this study are included within the article.
